# The neurogenic response of cardiac resident nestin^(+)^ cells was associated with GAP43 upregulation and abrogated in a setting of type I diabetes

**DOI:** 10.1186/1475-2840-12-114

**Published:** 2013-08-12

**Authors:** Andreanne Chabot, Marc-Andre Meus, Vanessa Hertig, Natacha Duquette, Angelino Calderone

**Affiliations:** 1 Montreal Heart Institute, Research Center, 5000 Belanger Street East, Montreal, QC H1T 1C8, Canada; 2 Département de Physiologie, Université de Montréal, Montreal, QC, Canada

**Keywords:** Myocardial infarction, Diabetes, Nestin^(+)^ cells, GAP43, Neurogenesis

## Abstract

**Background:**

Cardiac nestin^(+)^ cells exhibit properties of a neural progenitor/stem cell population characterized by the *de novo* synthesis of neurofilament-M in response to ischemic injury and 6-hydroxydopamine administration. The induction of growth associated protein 43 (GAP43) was identified as an early event of neurogenesis. The present study tested the hypothesis that the *de novo* synthesis of neurofilament-M by nestin^(+)^ cells was preceded by the transient upregulation of GAP43 during the acute phase of reparative fibrosis in the infarcted male rat heart. Secondly, a seminal feature of diabetes is impaired wound healing secondary to an inadequate neurogenic response. In this regard, an additional series of experiments tested the hypothesis that the neurogenic response of cardiac nestin^(+)^ cells was attenuated in a setting of type I diabetes.

**Methods:**

The neurogenic response of cardiac nestin^(+)^ cells was examined during the early phase of reparative fibrosis following permanent ligation of the left anterior descending coronary artery in the adult male rat heart. The experimental model of type I diabetes was created following a single injection of streptozotocin in adult male rats. The impact of a type I diabetic environment on the neurogenic response of cardiac nestin^(+)^ cells was examined during myocardial infarction and following the administration of 6-hydroxydopamine.

**Results:**

During the early phase of scar formation/healing, the density of GAP43/nestin^(+)^ fibres innervating the peri-infarct/infarct region was significantly increased, whereas neurofilament-M/nestin^(+)^ fibres were absent. With ongoing scar formation/healing, a temporal decrease of GAP43/nestin^(+)^ fibre density and a concomitant increase in the density of innervating neurofilament-M/nestin^(+)^ fibres were observed. The neurogenic response of cardiac nestin^(+)^ cells during scar formation/healing was inhibited following the superimposition of type I diabetes. The *de novo* synthesis of neurofilament-M by nestin^(+)^ cells after 6-hydroxydopamine administration was likewise attenuated in the heart of type I diabetic rats whereas the density of GAP43/nestin^(+)^ fibres remained elevated.

**Conclusion:**

The transient upregulation of GAP43 apparently represents a transition event during the acquisition of a neuronal-like phenotype and a type I diabetic environment attenuated the neurogenic response of cardiac nestin^(+)^ cells to ischemia and 6-hydroxydopamine.

## 

Sympathetic hyperinnervation of the viable myocardium and peri-infarct/infarct region represents a seminal feature of myocardial infarction [1-4]. Sympathetic fibres innervating the non-infarcted myocardium provide inotropic support to the ischemically damaged heart and contribute to the hypertrophic response [5,6]. During the reparative fibrotic response, innervating sympathetic fibres may directly contribute to scar formation/healing via the release of norepinephrine and subsequent stimulation of myofibroblast growth [7,8]. However, neural remodelling of the peri-infarct/infarct region was not limited to sympathetic fibre hyperinnervation as work from our lab reported the appearance of neuronal-like fibres originating from cardiac resident nestin^(+)^ cells [9,10]. The normal rodent heart contains a population of nestin^(+)^ cells that exhibit properties of a neural progenitor/stem cell [11-15]. *In vitro* experiments revealed that a subpopulation of cardiac resident nestin^(+)^ cells differentiated to a neuronal phenotype when plated in the appropriate induction milieu characterized by the upregulation of neurofilament-M and beta III-tubulin [12]. The *in vitro* response was recapitulated *in vivo* as the *de novo* synthesis of neurofilament-M by a subpopulation of cardiac resident nestin^(+)^ cells was observed during the reparative fibrotic response of the ischemically damaged rat heart and after 6-hydroxydopamine administration [9,10,12]. The induction of growth associated protein 43 (GAP43) was identified as an early event of neurogenesis in development and learning and increased expression was reported during the regeneration of injured peripheral nerves [16-19]. *In vitro* studies further revealed that the transition of skin-derived cells from a proliferative precursor to a neuronal phenotype was associated with the downregulation of nestin and concomitant expression of GAP43 [13]. Despite these findings, the expression and temporal regulation of GAP43 during neural progenitor/stem cell transition to a neuronal phenotype in a setting of wound healing remains unexamined. Therefore, the present study tested the hypothesis that the *de novo* synthesis of neurofilament-M by a subpopulation of cardiac resident nestin^(+)^ cells during the early phase of reparative fibrosis was preceded by the transient upregulation of GAP43.

It has been well established that diabetic patients have a higher mortality rate following acute myocardial infarction as compared to non-diabetic patients [20,21]. The latter paradigm was recapitulated experimentally as Luo and colleagues reported that the superimposition of myocardial infarction on type I diabetic mice led to 70-80% mortality 3–4 days following complete coronary artery ligation and was 2-fold higher than non-diabetic myocardial infarcted mice [22]. A pathological feature of diabetes that may contribute to the reported increase in mortality is impaired wound healing secondary to an inadequate neurogenic response [23-26]. The inadequate neurogenic response reported in diabetic injured tissue may be related in part to the suppression of neural/progenitor stem cell differentiation to a neuronal phenotype. In this regard, a second series of experiments tested the hypothesis that the transition of cardiac resident nestin^(+)^ cells to a neuronal-like phenotype in the ischemically damaged rat heart and after 6-hydroxydopamine administration was suppressed in a type 1 diabetic environment.

## Methods

### Myocardial infarction and type I diabetes

Myocardial infarction (MI) in adult male Sprague–Dawley rats (9–11 weeks old; Charles Rivers, Canada) was created by permanently ligating the left anterior descending coronary artery [10]. Sham animals underwent the identical surgical procedure except the coronary artery was not ligated. Experimental model of type I diabetes was induced following a single intraperitoneal injection of streptozotocin (60 mg/kg) in adult male Sprague–Dawley rats (9–11 weeks old; Charles Rivers, Canada) [27]. In a first study, adult rats were injected with streptozotocin and 1 week later subjected to permanent coronary artery ligation. This study was halted because of the high mortality rate of diabetic rats following surgery (please see Results section). An alternative approach was employed as a proof of concept and 3-day post-MI rats were injected with streptozotocin and sacrificed 7 days later. In this study, all diabetic post-MI rats survived the protocol. In parallel, post-MI rats were sacrificed 10 days after surgery. Lastly, seven days after the injection of adult male Sprague–Dawley with streptozotocin, type I diabetic rats (*n* = 5) were administered 6-hydroxydopamine hydrobromide (Sigma-Aldrich; IV injection of 100 mg/kg/day for 3 days) and sacrificed 2 days after completion of treatment [9,12]. In parallel, sham rats (*n* = 4) were administered 6-hydroxydopamine as described above and sacrificed 2 days after last injection. Plasma glucose levels were measured with a glucometer (Bayer; Model Ascensia Elite XL) from blood samples obtained from the tail vein. Left ventricular function was measured with a microtip pressure transducer catheter (model SPR-407, 2 F, Millar instrument, Houston Texas) and data analyzed with the program IOX version 1.8.9 (Emka Technologies; Falls Church, VA) [9]. At the end of each experiment, the heart was removed, weighed and stored in 2-methylbutane for immunofluorescence or separated into the left ventricle, right ventricle and scar, weighed, stored at −80°C and subsequently used for protein analysis. To determine scar area, the infarct region was traced, the outlined region visualized with the Olympus SZX7 microscope (Tokyo, Japan) and the area measured with data acquisition program Stream Basic (Olympus). The use and care of laboratory rats was according to the Canadian Council for Animal Care and approved by the Animal Care Committee of the Montreal Heart Institute.

### Western blot

Protein lysates prepared from the left ventricle of normal and diabetic rats and non-infarcted left ventricle and scar of non-diabetic and diabetic infarcted rats were subjected to SDS-electrophoresis, as previously described [27]. Antibodies used include the mouse monoclonal anti-nestin (1:500; Chemicon, Temicula, CA), the rabbit polyclonal GAP43 (1:500; Abcam, Cambridge, MA) and the mouse monoclonal anti-GAPDH (1:50,000; Ambion, Austin TX). Following overnight incubation at 4°C, the appropriate secondary antibody-conjugated to horseradish peroxidase (1:20,000, Jackson Immunoresearch, West Grove, PA) was added and bands visualized utilizing the ECL detection kit (Perkin Elmer). Films were scanned with Image J software® and the target protein signal was depicted as arbitrary light units normalized to GAPDH protein content.

### Immunofluorescence

The heart was excised, immersed directly in 2-methylbutane (temperature maintained at −80°C), and stored at −80°C. Immunofluorescence on cardiac tissue (cryostat sections of 14 μm thickness) was performed as previously described [9,27]. Antibodies employed include the mouse monoclonal anti-nestin (1:500; Chemicon), rabbit polyclonal anti-GAP43 (1:150; Abcam) and rabbit polyclonal anti-neurofilament-M (1:500; Chemicon). The nucleus was identified with TO-PRO-3 (InVitrogen; 1.5 μM; emission wavelength, 661 nm) staining. Secondary antibodies used were a goat anti-mouse IgG conjugated to conjugated to Alexa-546 (1:600; InVitrogen; emission wavelength, 570 nm) and a goat anti-rabbit IgG conjugated to Alexa-488 (1:800; InVitrogen; emission wavelength, 520 nm). In post-MI and diabetic post-MI rats, neurofilament-M/nestin and GAP43/nestin fibre densities (μm^2^; area occupied by the fluorescent signal; LSM image Browser) were assessed in the peri-infarct and infarct regions. The fluorescent signal (μm^2^) was normalized to the peri-infarct/infarct area (mm^2^) and calculated as the average of at least 4 distinct fields. We have published several papers employing the latter approach to assess neurofilament-M and nestin fibre density in the adult rat heart [9,12]. Neurofilament-M/nestin and GAP43/nestin fibre densities in sham and diabetic rats treated with 6-hydroxydopamine were measured exclusively in the left ventricle whereas fibres residing at the epicardium were not considered. Non-specific staining was determined following the addition of an isotype control antibody or the conjugated secondary antibody alone. Immunofluorescence was visualized with a 10×- or 63×-oil 1.4 NA DIC.

### Statistics

Data are presented as the mean ± S.E.M, and (*n*) represents the number of rats used per experiment. Cardiac morphology, contractile function, immunofluorescence, plasma glucose and protein data were evaluated by a one-way ANOVA (GraphPad InStat) and a significant difference determined by the Student-Newman-Keuls Multiple Comparisons post-hoc test. Scar weight and surface area were evaluated by a student’s unpaired t-test and a value of *P* < 0.05 considered statistically significant.

## Results

### Neurofilament-M and GAP43 immunoreactivity of nestin^(+)^ fibres innervating the scar of infarcted rat hearts

**Figure 1:**
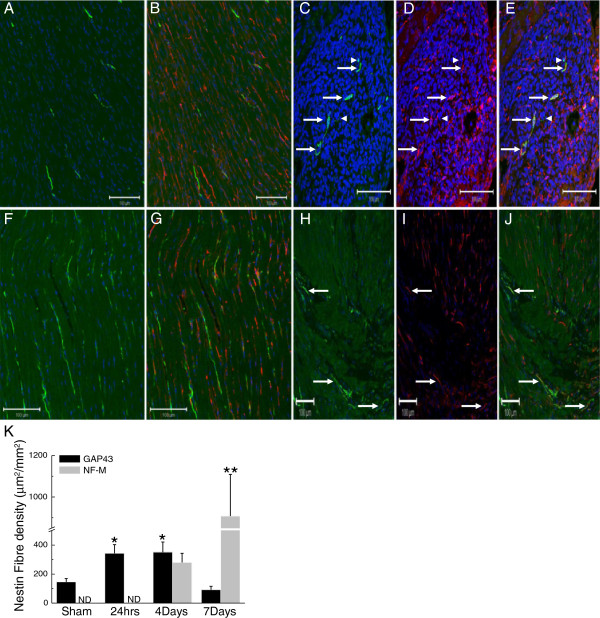
**Neurofilament-M and GAP43 expression by nestin**^**(+)**^ cells in the heart of sham and 1 week post-MI rat hearts. (Panels **A** &**B**) In the left ventricle of sham rats, neurofilament-M^(+)^ (green fluorescence) and nestin^(+)^ (red fluorescence) fibres were detected and co-expression of the intermediate filament proteins was not observed. (Panels **C**, **D** &**E**) In the peri-infarct/infarct region of a 4-day post-MI rat heart, innervating neurofilament-M^(+)^ fibres were detected and the preponderance physically associated with nestin^(+)^ fibres (indicated by arrow). Neurofilament-M^(+)^ fibres lacking nestin co-expression were also identified (indicated by arrowhead). (Panels **F** &**G**) GAP43^(+)^ (green fluorescence) and nestin^(+)^ (red fluorescence) fibres were detected in the normal rat heart and a paucity co-expressed both proteins. (Panels** H**, **I** &** J**) GAP43/nestin^(+)^ co-expressing fibres (indicated by arrow) were detected innervating the peri-infarct/infarct region 24 hrs after complete coronary artery ligation. The nucleus was identified with TO-PRO-3 staining (blue fluorescence). (Panel **K**) The density of GAP43/nestin^(+)^ fibres innervating the peri-infarct/infarct region was increased 24 hrs after myocardial infarction and preceded the appearance of neurofilament-M in nestin^(+)^ cells. With ongoing scar formation/healing, the density of GAP43/nestin^(+)^ fibres progressively decreased whereas a concomitant increase in neurofilament-M/nestin^(+)^ fibre density was apparent 4 and 7 days after myocardial infarction. (*) denotes *P* < 0.05 versus sham, (**) *P* < 0.05 versus 4 day infarcted rat hearts and (ND) not detected.

The normal adult Sprague–Dawley rat heart contains nestin^(+)^ cells intercalated among ventricular myocytes and neurofilament-M immunoreactive fibres innervating the myocardium (Figure 1A & B). The co-expression of the intermediate filament proteins neurofilament-M and nestin was not observed in the normal adult rat heart. Twenty-four hours following permanent coronary artery ligation of the adult heart, the ischemic region was apparent and innervated by nestin^(+)^ fibres. However, the co-expression of neurofilament-M in nestin^(+)^ fibres innervating the ischemic region of 1-day post-MI rats was not detected. By contrast, in the peri-infarct/infarct region of 4 day post-MI rat hearts, innervating nestin^(+)^ fibres co-expressing neurofilament-M were observed and neurofilament-M/nestin^(+)^ fibres persisted in the ischemic region of 7-day post-MI rat hearts (Figure 1C, D & E). GAP43-immunoreactive fibres were detected in the sham heart and a paucity of fibres co-expressed the intermediate filament protein nestin (Figure 1F & G). Twenty-four hours following complete coronary artery ligation of the adult rat heart, innervating GAP43- and nestin co-expressing immunoreactive fibres were observed (Figure 1H, I & J) and persisted in the peri-infarct/infarct region of 4 day post-MI rats. In the peri-infarct/infarct region of 7 day post-MI rat hearts, the appearance of GAP43/nestin^(+)^ fibres was markedly reduced. A quantitative analysis revealed that a population of neurofilament-M/nestin^(+)^ fibres were detected innervating the peri-infarct/infarct region of 4-day post-MI rats and a further significant increase in density was observed in 7-day post-MI rats (Figure 1K). By contrast, the density GAP43/nestin^(+)^ fibres innervating the peri-infarct/infarct region was increased in 1-day post-MI rats and remained significantly elevated in 4-day post-MI rats (Figure 1K) as compared to sham rats. In 7-day post-MI rats, the density of GAP43/nestin^(+)^ fibres innervating the peri-infarct/infarct region rats had returned to levels observed in the sham heart (Figure 1K).

### The impact of type I diabetes on neural remodelling of the scar in post-myocardial infarcted rats

Experimental type 1 diabetes was induced in adult male Sprague–Dawley rats following a single injection of streptozotocin (STZ; 60 mg/kg). One week later, the heart of type I diabetic rats (*n* = 23) underwent permanent coronary artery ligation. Within 24–48 hrs of coronary artery ligation, 70% (16/23 rats) of type I diabetic rats died. One week after surgery, 22% (5/23) of the surviving type I diabetic rats that underwent surgery did not have an infarct. Only 8% (2/23 rats) of type I diabetic rats that underwent permanent coronary artery ligation had an identifiable scar. No deaths were observed in sham rats injected with streptozotocin suggesting that the high mortality rate observed after coronary artery ligation was directly attributed to myocardial infarction. Unfortunately, the high mortality rate of type I diabetic rats after permanent coronary artery ligation precluded further continuation of experimental protocol. Therefore, as a proof of concept, type I diabetes was induced in 3-day post-MI rats. The 3-day post-MI time point was chosen as the appearance of neurofilament-M/nestin^(+)^ fibres was at an incipient stage during the acute phase of scar formation/healing (Figure 1K). In the MI group, 25% (6/24 rats) died within 24-48 hrs after coronary artery ligation. Interestingly, no deaths were observed 7 days after streptozotocin injection of 3-day post-MI rats despite the induction of a comparable hyperglycaemic environment as observed in diabetic rats that underwent coronary artery ligation. Left ventricular contractile function was significantly depressed in post-MI rats and diabetic rats as compared to sham rats (Table 1). Seven days following streptozotocin injection of 3-day post-MI rats, mean arterial pressure and left ventricular contractile indices were further decreased as compared to post-MI rats (Table 1). Despite the superimposition of type I diabetes to 3-day post-MI rats, scar weight and surface area were not significantly different as compared to post-MI rats (Table 1).

**Table 1:** Body weight, heart weight and hemodynamic parameters of sham, streptozotocin-treated (STZ), myocardial infarcted (MI) and streptozotocin-treated myocardial infarcted (MI + STZ) rats

	**Body weight (grams)**	**Heart weight (grams)**	**MAP (mmHg)**	**LVSP (mmHg)**	**LV + dP/dt (mmHg/sec)**	**LV-dP/dt (mmHg/sec)**	**Scar weight (grams)**	**Scar area (cm**^**2**^**)**	**Plasma glucose (mM)**
sham (*n* = 8)	350 ± 19	1.13 ± 0.05	126 ± 4	147 ± 5	6859 ± 2241	5832 ± 258	-	-	7 ± 1
STZ (*n* = 7)	315 ± 6	1.08 ± 0.06	88 ± 3^*^	112 ± 5^*^	6106 ± 322^*^	4144 ± 323^*^	-	-	33 ± 1*
MI (*n* = 7-8)	335 ± 8	1.14 ± 0.08	108 ± 3^*^	134 ± 5	5762 ± 172^*^	4392 ± 280^*^	0.055 ± 0.008	0.678 ± 0.11	8 ± 2
MI + STZ (*n* = 8-12)	266 ± 8^***^	0.932 ± 0.05***	81 ± 3^**^	97 ± 2^***^	4658 ± 115^***^	2935 ± 103^***^	0.058 ± 0.004	0.754 ± 0.06	29 ± 1^*^

MI indicates myocardial infarction; MAP, mean arterial pressure; LVSP, left ventricular systolic pressure; LV + dP/dt, rate of left ventricular contraction; LV -dP/dt, rate of left ventricular relaxation. Data are presented as mean ± S.E.M, evaluated by a Student’s unpaired t-test or a Newman-Keuls Multiple Comparisons post-hoc test, (*) represents *P* < 0.05 versus sham, (**) represents *P* < 0.05 versus sham and MI, (***) versus sham, MI and STZ and (*n*) number of rats examined.

**Figure 2:**
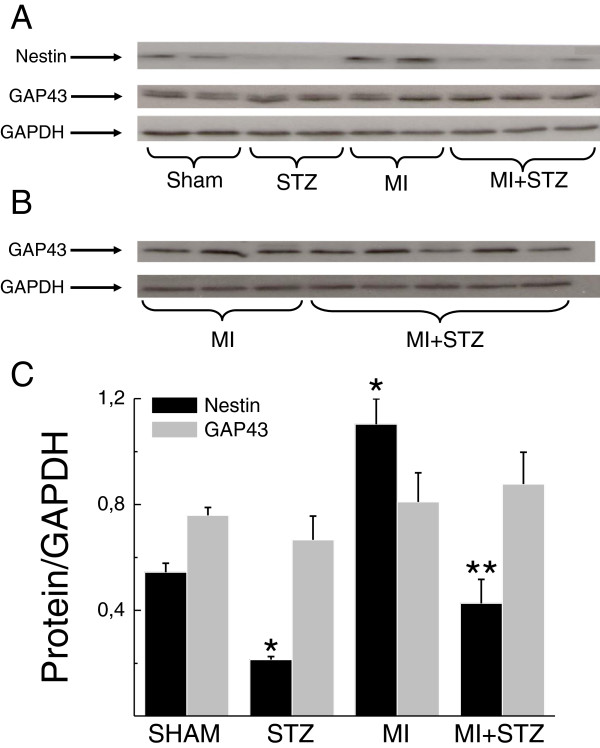
**Nestin and GAP43 protein expression in non-diabetic and diabetic infarcted rat hearts.** (Panel **A**). Nestin expression was increased in the non-infarcted left ventricle of myocardial infarcted (MI) rats. In streptozotocin (STZ) induced diabetic rats, nestin protein expression was diminished as compared to sham rats. Ventricular nestin protein levels remained reduced following the superimposition of type I diabetes on 3-day post-MI rats (MI + STZ) as compared to post-MI rats. Ventricular GAP43 protein levels were comparable in sham, diabetic, post-MI and diabetic post-MI rats. (Panel **B**) GAP43 expression was similar in the scar of myocardial infarcted (MI) and diabetic post-MI rats (MI + STZ). (Panel **C**) Semi-quantitative assessment of nestin and GAP43 expression in the left ventricle of sham (*n* = 4-5) and diabetic rats (STZ; *n* = 4) and the non-infarcted left ventricle of post-MI (MI; *n* = 4) and diabetic post-MI rats (MI + STZ; *n* = 6). Data was normalized to GAPDH protein expression, (*) denotes *P* < 0.05 versus sham and (**) *P* < 0.05 versus MI.

In the non-infarcted left ventricle of post-MI rats, nestin protein expression was significantly increased as compared to sham rats (Figure 2A & C). In the left ventricle of diabetic rats, nestin protein levels were markedly reduced as compared to sham rats (Figure 2A & C). In the non-infarcted left ventricle of diabetic post-MI rats, nestin protein levels were modestly increased as compared to diabetic rats but did not reach statistical significance. However, nestin protein levels in the non-infarcted left ventricle of diabetic post-MI rats remained significantly lower compared to post-MI rats (Figure 2A & C). Nestin protein content (normalized to GAPDH) was also reduced in the infarct region of diabetic post-MI rats (0.47 ± 0.11; *n* = 8; *P* < 0.05 versus non-diabetic post-MI rats) as compared to post-MI rats (0.83 ± 0.10; *n* = 6). By contrast, GAP43 protein levels in the heart of sham, diabetic, post-MI and diabetic post-MI rats were comparable (Figure 2A & C). Likewise, GAP43 protein expression in the scar of diabetic post-MI rats was similar to post-MI rats (Figure 2B).

**Figure 3:**
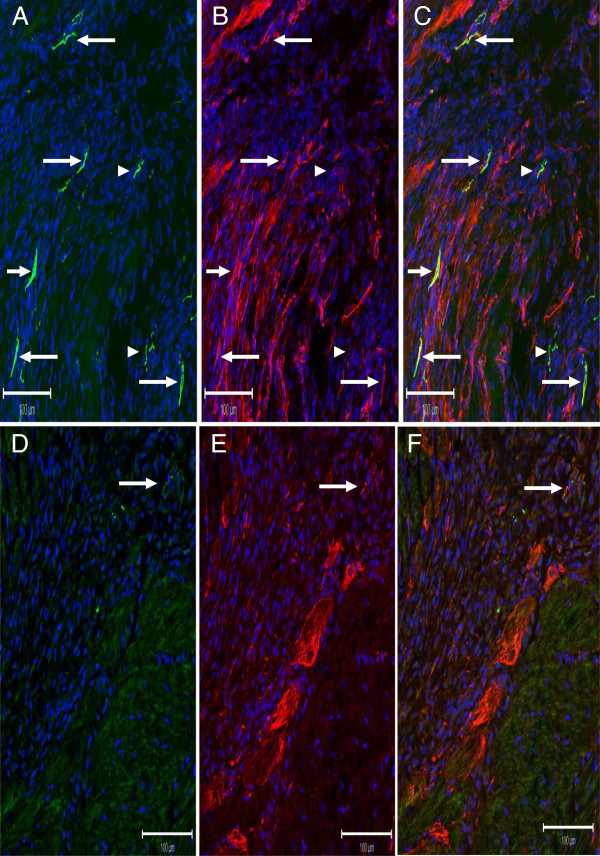
**The impact of type I diabetes on the neurogenic response of cardiac resident nestin**^**(+) **^**cells during scar formation/healing of the myocardial infarcted rat heart.** (Panels **A**, **B** &**C**) Neurofilament-M^(+)^ (green fluorescence) and nestin^(+)^ (red fluorescence) fibres were detected innervating the peri-infarct/infarct region of a post-MI rat heart and co-expression was evident by the emergence of a yellow fluorescence (indicated by arrow). Neurofilament-M^(+)^ fibres lacking nestin co-expression were also identified (indicated by arrowhead). (Panels **D**, **E** &**F**) The reparative fibrotic response of diabetic post-MI rats was associated with a paucity of innervating neurofilament-M/nestin^(+)^ fibres (indicated by arrow) in the peri-infarct/infarct region. Nestin^(+)^ cardiac myocyte-like cells were detected bordering the scar region and residual nestin^(+)^ cells and fibres were identified in the peri-infarct/infarct region.

In the heart of 10-day post-MI rats, the reparative fibrotic response was associated with neurofilament-M^(+)^ fibres (754 ± 126 μm^2^/mm^2^; *n* = 3) innervating the peri-infarct/infarct region and the majority co-expressed nestin (608 ± 79 μm^2^/mm^2^; *n* = 3) (Figure 3A, 3B &3C). In the heart of diabetic post-MI rats (Figure 3D, E & F), the density of neurofilament-M^(+)^ (244 ± 96 μm^2^/mm^2^; *n* = 3; *P* < 0.05 versus MI rats) and neurofilament-M/nestin^(+)^ fibres (71 ± 34 μm^2^/mm^2^; *n* = 3; *P* < 0.01 versus MI rats) innervating the peri-infarct/infarct region were significantly reduced as compared to post-MI rats. The residual expression of the intermediate filament protein in the infarct region of diabetic post-MI rats was attributed at least in part to the continued appearance of nestin^(+)^ cells with distinct processes and nestin^(+)^ cardiomyocyte-like cells (Figure 3E).

### The impact of type I diabetes on neural remodelling of the heart following 6-hydroxydopamine administration

**Figure 4:**
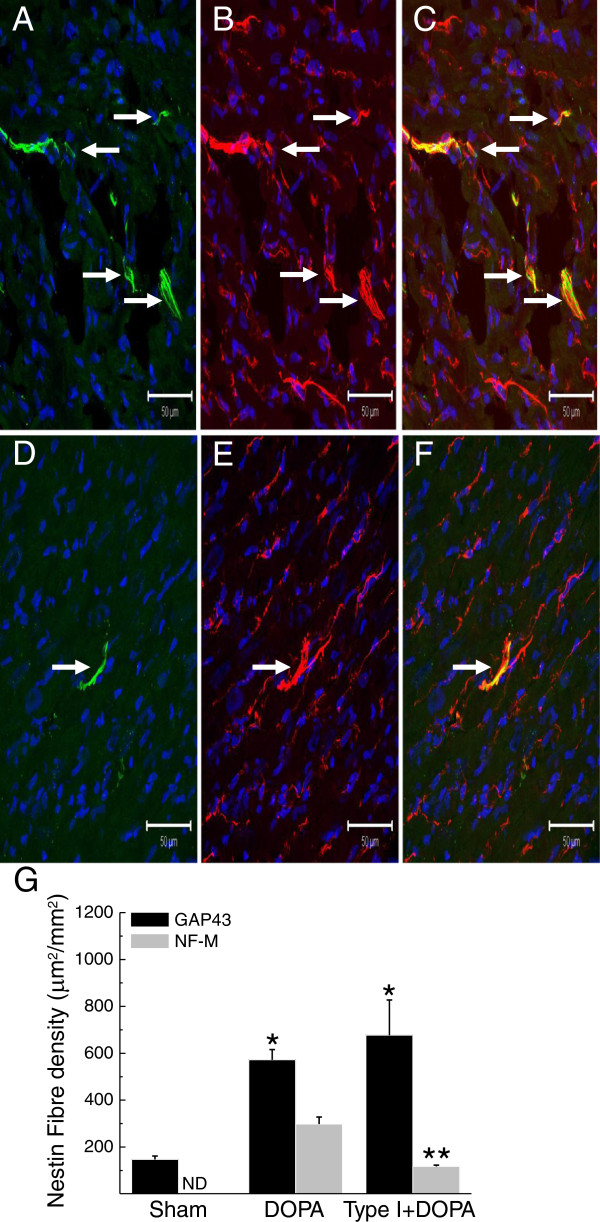
**The impact of type I diabetes on the neurogenic response of cardiac resident nestin**^**(+) **^**cells following administration of 6-hydroxydopamine.** (Panels **A**, **B** &**C**) Sham rats treated with 6-hydroxydopamine promoted the *de novo* synthesis of neurofilament-M (green fluorescence) by cardiac resident nestin^(+)^ (red fluorescence) cells and the intermediate filament proteins were physically associated as reflected by the emergence of a yellow fluorescence (indicated by arrow). (Panels **D**, **E**, &**F**) In type I diabetic rats, the *de novo* synthesis of neurofilament-M (green fluorescence) by cardiac resident nestin^(+)^ cells (red fluorescence) was attenuated in response to 6-hydroxydopamine. The nucleus was identified with TO-PRO-3 staining (blue fluorescence). (Panel **G**) The administration of 6-hydroxydopamine (DOPA) significantly increased the density of GAP43/nestin^(+)^ and neurofilament-M/nestin^(+)^ fibres in the heart of sham rats. In the left ventricle of type 1 diabetic rats treated with 6-hydroxydopamine (Type I + DOPA), the density of GAP43/nestin^(+)^ fibres remained elevated as compared to sham rats treated with 6-hydroxydopamine. By contrast, the *de novo* synthesis of neurofilament-M was partially inhibited as reflected by the significant reduction of neurofilament-M/nestin^(+)^ fibre density in the heart of type I diabetic rats treated with 6-hydroxydopamine. (*) denotes *P* < 0.05 versus normal, (**) *P* < 0.05 versus 6-hydroxydopamine-treated normal rats (DOPA) and (ND) not detected.

The present study has demonstrated that the superimposition of type I diabetes on MI rats attenuated the neurogenic response of nestin^(+)^ cells during reparative fibrosis. To complement and further support these data, additional experiments were performed to test the hypothesis that a prevailing type I diabetic environment would likewise suppress the differentiation of cardiac nestin^(+)^ cells to a neuronal-like phenotype in response to a neurogenic stimulus. Work from our lab has been previously demonstrated that 6-hydroxydopamine administration to normal adult rats promoted the *de novo* synthesis of neurofilament-M by cardiac nestin^(+)^ cells [9,12]. In this regard, 6-hydroxydopamine was administered to streptozotocin-injected rats with established hyperglycaemia. Type I diabetic rats were injected daily for a period of 3 days with 6-hydroxydopamine and sacrificed 2 days later. In 6-hydroxydopamine-treated rats (*n* = 4; plasma glucose = 7.9 ± 0.1 mM), GAP43/nestin^(+)^ and neurofilament-M/nestin^(+)^ fibres (Figure 4A, B & C) were detected innervating the left ventricle. Following 6-hydroxydopamine administration of type I diabetic rats (*n* = 5; plasma glucose = 26 ± 3.9 mM), GAP43/nestin^(+)^ fibres innervating the left ventricle persisted whereas neurofilament-M/nestin^(+)^ fibres (Figure 4D, E & F) were less apparent. A quantitative analysis revealed that the density of GAP43/nestin^(+)^ fibres in the heart of 6-hydroxydopamine-treated rats was increased as compared to sham rats and remained significantly elevated following 6-hydroxydopamine administration of type I diabetic rats (Figure 4G). The density of neurofilament-M/nestin^(+)^ fibres in the heart of 6-hydroxydopamine-treated rats was also significantly increased as compared to sham rats (Figure 4G). By contrast, a significant reduction in the density of neurofilament-M/nestin^(+)^ fibres innervating the myocardium was observed in 6-hydroxydopamine-treated type I diabetic rats as compared to 6-hydroxydopamine-treated rats (Figure 4G).

## Discussion

The rodent heart contains resident nestin^(+)^ cells that exhibit properties of a neural progenitor/stem cell population [9,11-15]. Consistent with this premise, the plating of cardiac-derived nestin^(+)^ cells in a defined induction milieu revealed that a subpopulation differentiated to a neuronal phenotype characterized by the loss of nestin expression and concomitant upregulation of neurofilament-M and beta III-tubulin [12]. The *in vitro* findings were reproduced *in vivo* as the *de novo* synthesis of neurofilament-M by a subpopulation of cardiac resident nestin^(+)^ cells and subsequent physical association of the intermediate filament proteins represents a seminal event of reparative fibrosis following ischemic injury of the adult rat heart [9,12].

### Transient upregulation of GAP43 by cardiac nestin^(+)^ cells during reparative fibrosis

Previous studies have identified growth associated protein 43 (GAP43) as an early and important mediator of neurogenesis in development and learning and increased expression was reported during the regeneration of injured peripheral nerves [16-19]. *In vitro* studies have further revealed that skin-derived cells downregulate nestin and concomitantly express GAP43 during a transition from a proliferative precursor to a neuronal phenotype [13]. Despite these findings, the expression and temporal pattern of GAP43 regulation during the transition of a neural progenitor/stem cell population to a neuronal phenotype during wound healing remains unexamined. Therefore, the present study tested the hypothesis that the *de novo* synthesis of neurofilament-M by a subpopulation of cardiac resident nestin^(+)^ cells during the early phase of reparative fibrosis was preceded by the transient upregulation of GAP43. In the normal adult rat heart, a paucity of GAP43^(+)^ fibres innervating the left ventricle co-expressed the intermediate filament protein nestin. Twenty-fours hours after complete coronary artery ligation, GAP43/nestin^(+)^ fibres were detected innervating the peri-infarct/infarct region of the ischemically damaged rat heart, remained elevated in 4 days post-MI rats and gradually returned to levels observed in sham rats 7 days after myocardial infarction. By contrast, the co-expression of nestin and neurofilament-M was not detected in the peri-infarct/infarct region of 1-day post-MI rats. However, with ongoing scar formation/healing, the temporal decrease of GAP43/nestin^(+)^ fibre density was associated with a concomitant increase in the density of innervating neurofilament-M/nestin^(+)^ fibres. Collectively, these data have demonstrated that GAP43 was upregulated in cardiac resident nestin^(+)^ cells prior to the *de novo* synthesis of neurofilament-M during wound healing of the ischemically damaged rat heart. The transient induction of GAP43 during the reparative fibrotic response apparently represents an intermediate event implicated in the transition of a subpopulation of cardiac nestin^(+)^ cells to a neuronal-like phenotype.

### High mortality rate of type I diabetic rats was observed following myocardial infarction

Numerous studies have identified a proliferative role of the intermediate filament protein nestin in various cell types including neural/progenitor stem cells [11]. An additional biological function was identified during axonal sprouting in skeletal muscle. Following muscle injury or in response to a constitutively active neuregulin receptor, newly formed nestin^(+)^ fibres were expressed by Schwann cells located at the motor endplate and physically associated with axonal sprouts growing from the nerve terminal [28,29]. The authors suggested that the emergence of nestin^(+)^ fibres from Schwann cells may directly support the growth of nerve sprouts possibly acting as a scaffold and/or guidance mechanism. As previously reported and documented in the present study, the reparative fibrotic response of the ischemically damaged rat heart was associated with the *de novo* synthesis of neurofilament-M by a subpopulation of cardiac resident nestin^(+)^ cells. Thereafter, newly formed neurofilament-M was physically associated with nestin^(+)^ fibres and innervated the peri-infarct/infarct region. Thus, it is tempting to speculate that the suppression of nestin could significantly compromise the neurogenic response of Schwann cells and/or neural progenitor/stem cells during tissue repair. Impaired wound healing represents an established pathophysiological feature of diabetes related in part to an inadequate neurogenic response [23-26]. In the heart of experimental rat models of type I and II diabetes, nestin protein levels were significantly reduced and not attributed to cellular apoptosis [27]. These data suggested that the downregulation of the intermediate filament protein in a setting of diabetes may significantly limited the neurogenic response of a subpopulation of cardiac resident nestin^(+)^ cells during reparative fibrosis. To directly examine the premise, streptozotocin-induced type I diabetic rats were subjected to complete coronary artery occlusion. In this initial study, a high mortality rate (70%) was observed 24–48 hrs following permanent coronary artery ligation of the heart of type 1 diabetic rats. An identical observation was reported by Luo and colleagues as permanent coronary artery ligation of the heart of type 1 diabetic mice led to a 2-fold greater increase in mortality within 3–4 days of surgery as compared to non-diabetic post-MI mice [22]. In our study, no deaths were reported in type I diabetic rats supporting that premise that the superimposition of myocardial infarction was directly responsible for the high mortality rate. These data and those reported by Luo and colleagues were consistent with clinical studies indicating that diabetic patients have a higher mortality rate following acute myocardial infarction and an increased risk of progression to heart failure post-infarction as compared to non-diabetic post-MI patients [20,21].

### The superimposition of type I diabetes on myocardial infarcted rats attenuated the neurogenic response of cardiac nestin^(+)^ cells

The high mortality rate of type I diabetic rats subjected to permanent coronary artery ligation precluded further continuation of the experimental model. Nonetheless, the experimental protocol was adapted and as a proof of concept, streptozotocin was injected in 3 day post-MI rats and sacrificed 7 days later. In contrast to that observed following the superimposition of myocardial infarction on type I diabetic rats, streptozotocin injection of 3 day post-MI rats did not lead to any deaths. As expected, the imposition of type I diabetes on post-MI rats exacerbated left ventricular dysfunction as compared to post-MI rats. In type I diabetic post-MI rats, nestin protein levels of the non-infarcted left ventricle and scar were significantly reduced as compared to post-MI rats. In post-MI rats, neurofilament-M^(+)^ fibres were detected innervating the peri-infarct/infarct region and the preponderance physically associated with nestin^(+)^ fibres. In the peri-infarct/infarct region of type I diabetic post-MI rat hearts, neurofilament-M^(+)^ and neurofilament-M/nestin^(+)^ fibre densities were significantly reduced. The inadequate neurogenic response in the scar of type I diabetic post-MI rats was apparently not secondary to a downregulation of GAP43 protein levels. Thus, the reduced neurogenic response in the scar of diabetic post-MI rats was attributed in part to the inadequate *de novo* synthesis of neurofilament-M by a subpopulation of cardiac resident nestin^(+)^ cells. Downregulation of the intermediate filament protein nestin may represent in part an underlying mechanism suppressing the transition of cardiac resident nestin^(+)^ cells to a neuronal-like phenotype. Moreover, we cannot exclude the possibility that the prevailing hyperglycaemic environment may have further inhibited the synthesis of neurofilament-M by cardiac nestin^(+)^ cells.

### The induction of a type I diabetic environment rather than a direct effect of streptozotocin attenuated the neurogenic response of post-MI rats

The uptake of streptozotocin in pancreatic beta cells is attributed to the presence of the GLUT2 transporter [30]. Following entry and accumulation in beta cells, the alkylating action of streptozotocin leads to toxicity, cell death and subsequent loss of insulin synthesis [30]. By contrast, insulin-producing cells that do not express GLUT2 are resistant to the direct pathological action of streptozotocin [30]. Moreover, organ damage reported in the kidney and liver following streptozotocin administration was likewise attributed to the uptake of the drug by the GLUT2 transporter [30]. Based on these data, the attenuated neurogenic response of cardiac nestin^(+)^ cells during reparative fibrosis following the superimposition of type I diabetes may have been related in part to a direct effect of streptozotocin. However, the GLUT2 transporter was not expressed in adult rat brain-derived neural stem cells whereas GLUT1 and GLUT3 were highly abundant and the GLUT2 transcript was undetectable in the adult rodent heart [31,32]. The tissue specific distribution of GLUT2 was consistent with the disparate level of radioactive-labeled streptozotocin detected in the liver and heart following intravenous injection of the adult male rat [33]. In addition, streptozotocin is a highly unstable molecule with a reported biological half-life of ~5 minutes and ~40 minutes in rodents and humans, respectively [34,35]. Therefore, based on the short half-life of streptozotocin and apparent absence of GLUT2 transporter expression in the rodent heart as well as neural progenitor/stem cells, it is highly unlikely that the compromised neurogenic response of cardiac nestin^(+)^ cells during reparative fibrosis was related to a direct effect of streptozotocin that persisted for 7 days.

### A type I diabetic environment attenuated the neurogenic response of cardiac nestin^(+)^ cells following 6-hydroxydopamine administration

To reaffirm that a hyperglycaemic environment rather than a direct effect of streptozotocin suppressed the neurogenic response of cardiac resident nestin^(+)^ cells, 6-hydroxydopamine was administered to rats with established type I diabetes. Work from our lab has previously demonstrated that the treatment of normal adult male rats with 6-hydroxydopamine led to the degeneration of sympathetic fibres and concomitantly induced the *de novo* synthesis of neurofilament-M by a subpopulation of cardiac resident nestin^(+)^ cells [9,12]. Therefore, the following experiments tested the hypothesis that the neurogenic response of nestin^(+)^ cells to 6-hydroxydopamine was compromised in the heart of type I diabetic rats. In the left ventricle of 6-hydroxydopamine-treated rats, the density of GAP43/nestin^(+)^ and neurofilament-M/nestin^(+)^ fibres were significantly increased as compared to non-treated rats. Seven days following streptozotocin injection, 6-hydroxydopamine was administered to type I diabetic rats and the density of GAP43/nestin^(+)^ fibres in the left ventricle remained significantly elevated and quantitatively similar to the response observed in 6-hydroxydopamine treated rats. By contrast, the density of neurofilament-M/nestin^(+)^ fibres in the left ventricle of 6-hydroxydopamine-treated diabetic rats was significantly reduced. Thus, in response to 6-hydroxydopamine, a type I diabetic environment abrogated the transition of cardiac resident nestin^(+)^ cells to a neuronal-like phenotype via the selective suppression of neurofilament-M synthesis. These data complement the paradigm identified in the heart of 3-day post-MI rats subjected to type I diabetes and further supports the premise that a diabetic milieu rather than a direct effect of streptozotocin compromised the neurogenic response of a subpopulation of cardiac resident nestin^(+)^ cells.

## Conclusion

The temporal disparity of GAP43 and neurofilament-M expression by cardiac resident nestin^(+)^ cells during scar formation/healing of the ischemically damaged rat heart suggests that the prior induction of GAP43 may represent an essential intermediate event in the transition to a neuronal-like phenotype. Inadequate wound healing represents a hallmark feature of diabetes related in part to an impaired neurogenic response. The present study has reaffirmed and further expanded on the latter paradigm as the superimposition or the pre-existence of a type I diabetic environment abrogated the neurogenic response of cardiac resident nestin^(+)^ cells to ischemia and 6-hydroxydopamine, respectively. These findings may not be limited to the rodent as nestin^(+)^ cells were identified in the human heart, brain and skin [9,12,36,37]. Moreover, at least in the human brain and skin, a subpopulation of nestin^(+)^ cells exhibited properties of a neural progenitor/stem cell population including neurosphere formation and differentiation to a neuronal cell [36,37]. In this regard, the inadequate differentiation of nestin^(+)^ cells to a neuronal phenotype may in part contribute to the impaired neurogenic response reported during wound healing of injured diabetic tissue.

## Competing interests

The authors declare that they have no competing interests.

## Authors’ contributions

AC generated animal models, performed Western blots and involved in drafting of the manuscript. MM: Immunofluorescence. VH: Immunofluorescence. ND: Animal models. AC: Design and coordination of the study and involved in the writing of the manuscript. All authors read and approved the final manuscript.
